# Role of Interleukin 17 in Severe Acute Pancreatitis: Human Retrospective Pilot Study

**DOI:** 10.3390/diseases14100351

**Published:** 2026-09-23

**Authors:** Diana Iosif, Viorel Gherghina, Andra Iulia Suceveanu, Cristian Ionut Orasanu, Marius Dragos Prazaru, Iulia Cindea, Anca Pantea Stoian, Laura Mazilu, Alina Doina Nicoara, Adrian Paul Suceveanu

**Affiliations:** 1 Doctoral School of Medicine, “Ovidius” University of Constanta, 900470 Constanta, Romania; 2 Department of Intensive Care Unit, “Sf. Apostol Andrei” Emergency County Hospital, 900591 Constanta, Romania; 3 Faculty of Medicine, “Ovidius” University of Constanta, 900470 Constanta, Romania; 4 Department of Gastroenterology, “Sf. Apostol Andrei” Emergency County Hospital, 900591 Constanta, Romania; 5 Clinical Service of Pathology, Departments of Pathology, “Sf. Apostol Andrei” Emergency County Hospital, 900591 Constanta, Romania; 6 Center for Research and Development of the Morphological and Genetic Studies of Malignant Pathology (CEDMOG), “Ovidius” University of Constanta, 900591 Constanta, Romania; 7 Department of Diabetes, Nutrition and Metabolic Diseases, “Carol Davila” University of Medicine and Pharmacy, 020021 Bucharest, Romania; 8 Department of Diabetes and Metabolic Diseases, “Prof. N.C. Paulescu” National Institute of Diabetes, Nutrition and Metabolic Diseases, 020475 Bucharest, Romania; 9 Department of Oncology, Ovidius Clinical Hospital, 905900 Constanta, Romania

**Keywords:** severe acute pancreatitis, IL-17, mortality, multiorgan dysfunction syndrome

## Abstract

**Background/Objectives**: Severe acute pancreatitis accounts for 20% of acute pancreatitis episodes and carries a substantial mortality risk. The systematic severity of the disease is driven by a massive cytokine release, with Interleukin-17 (IL-17) acting as a crucial modulator of the pro-inflammatory cascade. This study aimed to evaluate the prognostic value of serum IL-17 at admission and correlate it with multi-organ dysfunctions and clinical-biological outcomes in patients with severe acute pancreatitis. **Methods**: A retrospective analysis was conducted on a cohort of 35 patients with severe acute pancreatitis. Demographic, etiological, and clinical-biological baseline characteristics were extracted. Serum IL-17 levels were determined at admission using the ELISA method, and data were statistically analyzed. **Results**: Significantly higher levels of IL-17 were observed in patients who developed multi-organ failure. Prognostic thresholds were identified via ROC analysis to predict organ dysfunctions: ≥19.77 pg/mL for neurological dysfunction (AUC = 0.814, *p* = 0.003) and acute respiratory distress syndrome (AUC = 0.756, *p* = 0.024); ≥18.45 pg/mL for respiratory (*p* = 0.004) and cardiovascular dysfunctions (AUC = 0.820, *p* = 0.002); and ≥19.81 pg/mL for renal dysfunction (AUC = 0.735, *p* = 0.020). Furthermore, univariate binary logistic regression identified elevated serum IL-17 as a significant prognostic factor for mortality among patients admitted to the intensive care unit (OR = 1.062, *p* = 0.048). **Conclusions**: Serum IL-17 at admission functions as a significant prognostic biomarker that reflects the severe systemic pro-inflammatory status in severe acute pancreatitis and mirrors the risk of subsequent organ dysfunctions and mortality. Incorporating IL-17 into initial risk stratification protocols may assist clinicians in timely therapeutic escalation.

## 1. Introduction

Acute pancreatitis is an inflammatory process where the erratic activation of trypsin causes its autodigestion. In approximately 20% of pancreatitis episodes, the evolution to severe acute pancreatitis occurs. According to the revised Atlanta classification, the condition is defined as the persistence of organ failure for more than 48 h, often associated with local and systemic complications. Most of them present foci of necrosis, an aspect that leads to a mortality of 15–30% of cases [1,2].

The etiology is varied, including gallstones, alcohol consumption, trauma (including iatrogenic), metabolic causes (hypertriglyceridemia, hypercalcemia), genetics, medication, or infections. Of these, the first two are most frequently involved, each representing between 30% and 50% [3,4]. Lithiasis is responsible for the occlusion of the ampulla of Vater and the increase in pressure in the bile duct. High pressures lead to abnormal calcium entry into acinar cells. At the same time, calcium is released from the endoplasmic reticulum, reducing the reserves stored in it. Bridges are formed between the endoplasmic reticulum and the plasmalemma, which will allow extracellular calcium to replenish its reserves. However, the uncontrolled entry of calcium into the cell results in a cytosolic load that covers the mitochondria. The major consequence is a decrease in adenosine triphosphate (ATP) production. Normally, there are mechanisms for regulating calcium concentration, but with a lower amount of ATP, they become ineffective. This leads to an exacerbation of calcium toxicity. The same intracellular mechanism is found in the case of ethyl esters (alcoholic etiology of pancreatitis) and triglyceride lipolysis (hypertriglyceridic etiology). The decrease in ATP production leads to defective autophagy, zymogen activation, cytoskeleton alteration, inflammasome activation, cytokine release, and cell necrosis [5,6,7,8,9].

The etiopathogenesis cascade leads to premature activation of trypsinogen and activation of the nuclear factor-kappa B (NF-κB) pathway in acinar cells, resulting in inflammation of the pancreas. The inflammatory substrate potentiated by cytokine release is responsible for the progression of severity and the stimulation of a pronounced immune response. Interleukin 17 (IL-17) is a proinflammatory cytokine produced by T helper 17 lymphocytes and natural killer cells. Its role is to mediate responses to pathogens. It also provides a balance between the inflammatory response and the immune system through the cytotoxic and tolerant balance, resulting in its proinflammatory status. IL-17A, a member of the IL-17 family, interacts with acinar cells, immune infiltrating cells, and other cytokines, being involved in the progression of acute pancreatitis [3,10,11].

Multidimensional profiling shows that IL-17 operates synchronously with IL-6, IL-1β, and tumor necrosis factor alpha (TNF-α) in triggering systemic inflammation. At the tissue level, experimental rat models confirm that local IL-17A directly causes acinar necrosis and histopathological lesions [12,13]. Inhibition of retinoid orphan receptor gamma t (RORγt) in vivo in cerulein-induced pancreatitis reduces Th17 and γδ-T cells and ameliorates pancreatic injury. Moreover, the decline of soluble fibrinogen-like protein 2 (sFGL2) levels in severe models of taurocholate-induced is associated with a severe imbalance between Th17 and regulatory T cells, which further exacerbates the destructive cascade [14,15]. Visnagin ameliorates the effects by modulating the Nrf2/NF-κB axis and decreasing IL-17 and multiorgan dysfunction in a dose-dependent manner, while β-elemene inhibits local inflammation and severity of pancreatitis by blocking the IL-17RA receptor on macrophages [16,17].

The current literature provides insufficient data regarding the involvement of IL-17 in the evolution of severe acute pancreatitis. The objective of the present research is to analyze this interleukin in the context of severe forms, aiming to identify potential implications by corroborating with the clinico-biological status and prognosis of patients.

## 2. Materials and Methods

### 2.1. Patient Selection and Study Design

We conducted a retrospective study focusing on patients admitted to the Emergency County Clinical Hospital in Constanta, Romania, from October 2024 to August 2025, specifically within the gastroenterology and intensive care units. The inclusion criteria consisted of patients who presented in the emergency room, were over 18 years old, and were diagnosed with severe acute pancreatitis. Exclusion criteria included minor patients, patients who did not have all the necessary tests, other forms of pancreatitis, and those who came by transfer between departments.

### 2.2. Clinical and Biological Profile

Diagnosis of severe acute pancreatitis was based on presence of local complications and/or persistent organ failure. The Balthazar D or E computed tomography score was the main tool used. The RANSON, APACHE, Imrie-Glasgow, BISAP, SOFA and SAPS3 prediction scores were also used. The hospitalized patients underwent daily monitoring of laboratory biological markers and were assessed for any changes in their condition. The biological samples analyzed correspond to the initial laboratory evaluations (baseline), being collected immediately upon patient admission, before the initiation of any therapeutic intervention. The markers included the hemoleukogram, Aggregate Index of Systemic Inflammation (AISI) score, erythrocyte sedimentation rate (ESR), C-reactive protein (CRP), serum amylase, serum lipase, calcium, blood glucose, triglycerides, pH, bicarbonate, and lactate. An increase in the number of white blood cells (leukocytosis) was defined as a total white blood cell count exceeding 10.0 × 10^3^/µL. The ranges used to define metabolic disorders were: hypocalcemia (total serum calcium < 8.5 mg/dL), hyperglycemia (fasting blood glucose 126 mg/dL), and hypertriglyceridemia (serum triglycerides > 200 mg/dL). Demographic data, including sex, and age, as well as etiological data—such as biliary, ethanolic, metabolic, and post-interventional causes—were collected based on observation sheets; pre-existing comorbidities (including respiratory, cardiovascular, digestive, metabolic, neurological, or oncological diseases); and clinical examinations. The duration of hospitalization, the length of stay in intensive care, the complications that arose (including pancreatic abscesses, infected pancreatic necrosis, ascites, and pleurisy), and the instances of organ dysfunction (such as pulmonary, cardiovascular, and renal issues) were recorded. The analyzed complications were diagnosed on the basis of standardized imaging and clinical criteria. Contrast-enhanced computed tomography showed infected pancreatic necrosis and pancreatic abscess. Diagnostic imaging (ultrasound, chest radiography) showed pathological fluid accumulation in the abdomen and thorax (ascites and pleurisy).

### 2.3. Serum Levels of IL-17

In order to determine IL-17 concentrations, peripheral blood samples were collected by venipuncture in sterile tubes immediately after the patient’s admission to the Gastroenterology department. The sampling was performed after the patient was transferred from the Emergency Department, strictly within the first 24 h from the onset of symptoms and prior to the initiation of any treatment. The serum was centrifuged at 1000× *g* for 20 min in a Thermo Scientific SL16R centrifuge (Thermo Fisher Scientific, Waltham, MA, USA). After extraction, the serum samples were then divided into aliquots and stored at −80 °C until analysis. An IL-17 ELISA kit was used (ELISAKit ELK2610, ELK Biotechnology, Wuhan, China). The applied test principle is the sandwich immunoenzymatic assay. The microtiter plate was pre-coated with an antibody specific for IL-17. The samples were added to the corresponding wells of the microtiter plate and then to a biotin-conjugated antibody specific for IL-17. Avidin conjugated to horseradish peroxidase is added to each well and incubated. After the addition of the 3,3′,5,5′-tetramethylbenzidine (TMB) substrate solution, only the wells with IL-17, biotin-conjugated antibody, and enzyme-conjugated avidin showed a color change. The enzyme-substrate reaction was terminated by the addition of a sulfuric acid solution. Color changes are measured spectrophotometrically at a wavelength of 450 nm ± 10 nm. The concentration of IL-17 in the samples was determined by comparing the optical density of the samples with a standard curve made based on seven human IL-17 standards and the blank sample, with a detection limit of <5.5 pg/mL for IL-17. The intra-assay and inter-assay precisions are CV% < 8% and CV% < 10%, respectively. No significant cross-reactivity or interference with the IL-17 fragment or analogs was observed.

### 2.4. Statistical Analysis

Statistical analysis was performed using SPSS Statistics Version 26 (IBM Corporation, Armonk, NY, USA). Indicators of central tendency and variability were used. Levene’s test was used to assess group homogeneity and to be able to use the most accurate tests, such as the Mann–Whitney U test and the Kruskal–Wallis H test for continuous variables and Fisher’s exact test for categorical data. Data association was performed using Pearson correlation. To evaluate the precision of the parameters, a receiver operating characteristic (ROC) curve and an area under the curve (AUC) analysis were used. The optimal decision threshold (cut-off) for sensitivity and specificity was set at the value that maximizes the AUC value. Odds ratio (OR) assessment was performed using binary logistic regression or linear regression tests, depending on the nature of the data. The statistical significance threshold was set at a *p* value ≤ 0.05.

### 2.5. Ethical Aspects

The study was conducted with favorable approval by the Local Ethics Committee for Clinical Studies and Developmental Research, and written informed consent was obtained from all participants before collection of biological samples (no. 46159/11 August 2023).

## 3. Results

We identified a group of 35 cases of severe acute pancreatitis. The median age was 64 years, and most were male. The most frequent comorbidities were represented by digestive and cardiovascular disorders. The most frequent etiologies were ethanol and biliary. In addition to the CT score that made it possible to classify the severity class, the RANSON, IMRIE, BISAP, SOFA, APACHE, and SAPS3 risk scores were also used (Table 1).

The biological profile of the study cohort on admission was marked by a severe inflammatory and metabolic status. Leukocytosis was present in 80% of the patients, with a median leukocyte count of 19.45 × 10^3^ µL (IQR: 14.62–25.98), and increased inflammatory markers: median ESR of 50.08 mm/h (IQR: 29.5–72.15) and CRP of 24.40 mg/dL (IQR: 14.08–30). At the same time, the calculated AISI score had a median value of 4216 (IQR: 2487–6384). Pancreatic enzymes were significantly elevated, with amylase elevated in 88.57% (median 823 U/L, IQR 300–1655) and lipase in 91.43% (median 1379.46 U/L, IQR 300–2194.80). Metabolic and acid-base disturbances were hypocalcemia (71.43% of patients), hyperglycemia (20% of patients), hypertriglyceridemia (14.29% of patients), a median pH of 7.37 (IQR: 7.26–7.41), a bicarbonate of 18 mmol/L (IQR: 14–23.9), and a median elevated lactate of 3.02 mmol/L (IQR: 2.24–4.37).

The median value of IL-17 was 19.03 pg/mL (16.87–25.56). Its increased values were directly associated with the severity scores IMRIE (r = 0.363, *p* = 0.032), BISAP (r = 0.336, *p* = 0.049), SOFA (r = 0.387, *p* = 0.022), and APACHE (r = 0.379, *p* = 0.025). Also, the increased values were associated with increased leukocytes (r = 0.646, *p* < 0.001) and lactate (r = 0.524, *p* = 0.006) and low bicarbonate (r = −0.534, *p* = 0.004).

The patients had a median hospitalization period of 9 days (5–12), and for 60% of them, intensive care was required. In intensive care, they had a median hospitalization period of 4 days (2.5–9.5 days). The overall in-hospital mortality rate was 34.29%, and the intensive care mortality rate was 57.14%. The most common complications were ascites, pleurisy, and organ dysfunction (Table 2). Almost a quarter of all patients developed acute respiratory distress syndrome (25.71%). In patients with diabetes, statistically significant correlations were identified with complications such as pancreatic abscesses (*p* = 0.002) and infected pancreatic necrosis (*p* = 0.038).

Higher IL-17 levels were found in patients with neurological dysfunction (*p* = 0.002) and in patients with cardiovascular (*p* = 0.001), respiratory (*p* = 0.004), and renal (*p* = 0.020) dysfunctions. IL-17 levels were higher in patients with acute respiratory distress syndrome and those with vasoactive support (*p* = 0.023 and *p* = 0.001, respectively).

To assess the prognostic value of serum IL-17 at admission, a ROC curve analysis was performed to predict specific systemic complications and organ dysfunction. The biomarker demonstrated strong predictive performance across all endpoints. Specifically, a cut-off value of IL-17 ≥ 19.77 pg/mL predicted the occurrence of neurological dysfunction (AUC = 0.814, *p* = 0.003, sensitivity 90.9%, specificity 75.0%) and ARDS (AUC = 0.756, *p* = 0.024, sensitivity 88.9%, specificity 69.2%). A cut-off value ≥ 18.45 pg/mL was predictive of both respiratory dysfunction (AUC = 0.797, *p* = 0.004, sensitivity 91.7%, specificity 65.2%) and cardiovascular dysfunction (AUC = 0.820, *p* = 0.002, sensitivity 92.9%, specificity 71.4%), while also reflecting the subsequent need for vasoactive support (AUC = 0.820, *p* = 0.002, sensitivity 92.9%, specificity 71.4%). Finally, a threshold of ≥19.81 pg/mL predicted renal dysfunction (AUC = 0.735, *p* = 0.020, sensitivity 78.6%, specificity 66.7%). Clinically, these identified cut-off values (ranging narrowly between 18.45 and 19.81 pg/mL) represent a critical biological threshold. Reaching or exceeding these serum levels indicates a hyperinflammatory state at baseline, driven by IL-17-mediated neutrophil recruitment and endothelial injury. Consequently, these specific thresholds serve as reliable early predictors of increased endothelial permeability and subsequent multiorgan failure in the course of acute pancreatitis.

Regarding the prognostic indicators that IL-17 can provide in relation to systemic dysfunctions, the following were highlighted: OR = 1.107 for neurological dysfunction (*p* = 0.028, CI95 1.011–1.212), OR = 1.098 for respiratory dysfunction (*p* = 0.036, CI95 1.006–1.198), OR = 1.114 for cardiovascular dysfunction (*p* = 0.027, CI95 1.012–1.226), and OR = 1.114 for the need for vasoactive support (*p* = 0.027, CI95 1.012–1.226).

Regarding aspects related to death, an increased value of IL-17 correlated with death (*p* = 0.037). Moreover, an IL-17 value of ≥19.23 pg/mL has a sensitivity of 83.3% and a specificity of 69.6% in predicting death (AUC = 0.717, *p* = 0.037). Therefore, univariate analysis of IL-17 in patients admitted to intensive care identifies it as a significant prognostic factor for death (OR = 1.062, *p* = 0.048, CI95 1.001–1.128).

## 4. Discussion

Our main results indicate a major prognostic role of IL-17 in the spectrum of organ dysfunctions, determining the complex systemic inflammatory response generated by severe acute pancreatitis. Th17 lymphocytes, the main source of IL-17, potentiate the inflammatory cascade by stimulating neutrophil infiltration and macrophage recruitment to the site of injury. Specifically, IL-17 amplifies this cascade by inducing granulocyte colony-stimulating factors and increasing the production of IL-8 by innate and epithelial immune cells. This triggers a massive migration of neutrophils—the first cells to infiltrate the inflamed pancreatic tissue—which release cytotoxic and oxidant mediators that destroy local acinar cells and cause damage to distant organ systems [18] (Figure 1).

Our results showing increased IL-17 on admission as a significant predictor of multiorgan dysfunction are in line with experimental models. In vivo studies indicated that the modulation of the Nrf2/NF-κB pathway to repress IL-17 expression could effectively arrest the related multiorgan dysfunction syndrome. This association confirms our statistics that the IL-17-driven hyperinflammatory status is a major systemic marker of remote organ failure in patients in intensive care [16,17].

One of the less common dysfunctions in our study group was neurological, manifested by seizures, psychomotor agitation, and cognitive dysfunction. Neurological involvement in severe acute pancreatitis includes a wide spectrum of acute or progressive manifestations that can evolve into pancreatic encephalopathy [19]. According to the meta-analysis by Meng Y. et al., neurological dysfunction can reach up to 33.33% of SAP, with mortality rates ranging between 10% and 87.50% [20]. Importantly, our univariate analysis found that serum IL-17 was a significant prognostic factor for these neurological deficits with an OR of 1.107 (*p* = 0.028). This statistical association reflects the role of IL-17 in the pathophysiology of injuries of the central nervous system.

In brain injury, IL-17 is associated with blood–brain barrier (BBB) disruption, immune cell recruitment, potentiation of inflammation, and direct destructive effect on neurons [21,22]. BBB disruption involves microglial activation, IL-6, MIP2, adhesion molecules, and nitric oxide production, neutrophil chemotaxis, and enzymatic barrier destruction. IL-17 also induces reactive oxygen species, upregulates endothelial adhesion molecules, and promotes migration of inflammatory cells [23,24]. Another characteristic of this cytokine, which reflected the presence of seizures in our group, is that it can cross the BBB and stimulate neuronal excitability. This mechanism involves stimulating glutamate release from astrocytes and inhibiting gamma-aminobutyric acid release, which increases neuronal excitotoxicity and inhibits synaptic transmission [22]. In addition, the long-term effect of IL-17 is implicated in chronic brain damage, such as Parkinson’s or Alzheimer’s disease [21,25]. This aspect makes IL-17 monitoring at the time of hospitalization provide double information. Firstly, it shows the prognostic risk of developing acute neurological dysfunction through the identified threshold value, and secondly, it provides insight into future neurodegenerative diseases that may develop.

In the present study, IL-17 emerged as a significant prognostic factor for predicting respiratory dysfunction, resulting in an OR of 1.098 (*p* = 0.036). Furthermore, a cut-off of IL-17 ≥ 19.77 pg/mL had a sensitivity of 88.9% and a specificity of 69.2% for prediction of ARDS (AUC = 0.756, *p* = 0.024). The basis of respiratory dysfunction is acute lung injury, and its severe form, ARDS, develops due to pulmonary inflammation and cell apoptosis [26]. ARDS is characterized by a rapid onset of impaired gas exchange leading to hypoxemic respiratory failure; it affects approximately 10% of patients admitted to intensive care units, and mortality rates are as high as 40–50% [27,28].

The precise role of IL-17 in these diseases is not well defined, but elevated levels are seen in infection-induced lung injury or sepsis-induced ARDS, leading to accumulation of inflammatory cells, dysfunction of the alveolar-capillary barrier, and a severe cytokine cascade [26]. In acute lung injury, activated alveolar macrophages generate pro-inflammatory mediators, and IL-17 enhances epithelial permeability and intrapulmonary neutrophil recruitment, leading to loss of endothelial integrity. In this way it changes the alveolar-arterial oxygen gradient, causes interstitial pulmonary edema, and activates the TLR7/IL-23/IL-17 pathway, which perpetuates the long-term inflammatory process [28]. IL-17 is also involved in the pro-inflammatory state observed in chronic respiratory diseases such as asthma, chronic obstructive pulmonary disease, bronchiectasis, or idiopathic fibrosis [29]. This complex mechanism is common to the IL family: IL-17A induces goblet cell metaplasia and mucus production, IL-17E drives allergic responses, IL-17C promotes autocrine inflammation and T-helper recruitment, and IL-17F induces pro-fibrotic factors that cause airway remodeling [30,31]. Similar to neurological damage, the implications of IL-17 in pulmonary dysfunction have an extended temporal resonance that warrants dynamic clinical monitoring.

Cardiovascular dysfunction in severe acute pancreatitis is due to aggressive fluid therapy or systemic inflammation. This condition can manifest as myocardial injury, repolarization changes secondary to hypocalcemia, decreased stroke volume, or pericardial effusions [32]. Elevated IL-17 levels were a significant predictor of cardiovascular dysfunction and need for vasoactive support in our study (OR = 1.114, *p* = 0.027). At the cardiac level, IL-17 induces cardiomyocyte apoptosis by activating inducible nitric oxide synthase. The IL-17A fraction causes ventricular remodeling and post-infarction dysfunction by activating fibroblasts [33]. IL-17 also triggers leukocyte activation, resulting in systemic leukocytosis, an association confirmed in our cohort. The influx of myocardial lymphocytes further causes structural and functional cardiac remodeling, leading to heart failure [34]. At the vascular level, the pro-inflammatory status generated by IL-17 accelerates the atherosclerotic process and causes collagen secretion by adventitial fibroblasts [33]. At the vascular level, IL-17 promotes atherosclerosis, instability of coronary artery plaques, and collagen secretion by adventitial fibroblasts [35]. This fibroblast activation leads to a decrease in vascular compliance and to large pressure variations [33,36].

Changes in vascular pressure often require vasoactive support to increase peripheral resistance and maintain tissue perfusion [37]. We found that high levels of IL-17 were an important prognostic factor for the prediction of the need for vasoactive therapy in our cohort (OR = 1.114, *p* = 0.027). Up to 80% of severe cases may require vasopressors, but early or high-dose use is associated with increased mortality. This phenomenon is attributed to severe vasoconstriction inducing visceral hypoperfusion (e.g., hepatic or extremity) and adverse cardiac events, such as ischemia or arrhythmias, in an already compromised myocardium [37,38].

Renal dysfunction was the most common dysfunction identified. In our cohort, higher levels of IL-17 at admission were a significant prognostic marker for the prediction of acute kidney injury (AKI) with a cut-off value of ≥19.81 pg/mL, which showed a significant predictive performance (AUC = 0.735, *p* = 0.020). Septic and ischemic phenomena are among the causes of AKI in severe acute pancreatitis, in which inflammatory cells may aggravate tissue damage or help repair. Specifically, Th17 lymphocytes producing IL-17 promote renal injury, and regulatory T cells repair the tissue [39]. In pancreatic sepsis, peripheral blood mononuclear cells cause increased systemic IL-17, while the associated systemic inflammatory response leads to abdominal compartment syndrome, hypovolemia, and intra-abdominal hypertension, severely compromising renal perfusion [40,41]. In particular, Th17 cells are the primary population of inflammatory cells that infiltrate the kidney during the first three days after an ischemic event [39]. At the renal epithelial level, IL-17 regulates blood pressure by modulating cellular sodium transporters. In the renal microvasculature, it induces small-caliber arterial remodeling and smooth muscle hypertrophy [42]. These factors, in addition to interstitial fibrosis, atherosclerotic lesions, and IL-17-mediated pericyte activation, create a vicious cycle that leads to further reduction in renal perfusion during ischemic events [42,43].

The most common complications encountered in our cohort were hematological. In severe acute pancreatitis, inflammation and coagulation share critical common pathways, driven by systemic capillary stasis and hypoxia-induced microcirculatory changes [44]. Acinar cells secrete cytokines, chemokines, and adhesion molecules into the systemic circulation, which trigger coagulation and recruitment of immune cells. This process leads to platelet aggregation, intravascular microthrombi, and fibrin deposition that can evolve into disseminated intravascular coagulation, a major contributor to persistent organ failure and mortality [45]. Microrheological changes secondary to acid-base and pH imbalances exacerbate these thromboinflammatory events [46]. Our findings provide strong support for this mechanism. Our patient cohort had significant metabolic and acid-base derangements on admission, including a median pH of 7.37, low bicarbonate, and high lactate. Also, our correlation analysis showed a direct association between elevated serum IL-17 levels and these severe metabolic abnormalities, with a significant positive correlation with increased lactate (r = 0.524, *p* = 0.006) and a negative correlation with decreased bicarbonate levels (r = −0.534, *p* = 0.004). The main effectors of this thromboinflammatory cascade are tumor necrosis factor, IL-1β, IL-6, and IL-17 that activate platelets and endothelial cells to enhance prothrombotic properties [47]. In particular, the IL-17A fraction independently stimulates thrombosis, inhibits anticoagulant factors, induces endothelial cell apoptosis, and upregulates adenosine diphosphate to enhance von Willebrand factor production [33]. In particular, the IL-17A fraction independently stimulates thrombosis, inhibits anticoagulant factors, induces endothelial cell apoptosis, and upregulates adenosine diphosphate to enhance von Willebrand factor production [48].

The main advantages of the present study are the focus on the prognostic role of serum IL-17 in predicting multiorgan dysfunction after severe acute pancreatitis and the comprehensive presentation of the mechanisms linking this interleukin to the acute systemic inflammatory process and its long-term effects. To date, most studies have focused on pancreatic tumor pathology in relation to IL-17. In this regard, an affinity of pancreatic cancer cells for interleukin has been observed. It represents a promoter and a supporter of the oncogenic process [49,50].

Several limitations need to be considered to interpret the results of this study. A major limitation is the small number of study participants. While statistically significant correlations and prognostic thresholds were successfully identified, the limited sample size from a single medical center limits the generalizability of our results to larger patient populations and may limit statistical power for less prevalent complications. The present study is also limited by its observational and retrospective design, which inherently bears the risk of selection bias and does not allow establishing definite causal relationships between IL-17 dynamics and organ failure. In addition, external validation of the identified threshold values in larger patient cohorts is needed. Future prospective multicenter studies are needed to validate these preliminary prognostic thresholds.

## 5. Conclusions

The present study demonstrates that serum IL-17 levels measured at admission reflect the intense systemic proinflammatory state generated during the course of severe acute pancreatitis. High IL-17 levels are not a cause but a useful prognostic biomarker correlating with severity of organ dysfunction and adverse clinical outcomes, including mortality in the intensive care unit. The results indicate that the addition of IL-17 to the initial laboratory workup may be beneficial in early risk stratification. Further prospective multicenter studies are needed to confirm its clinical utility in combination with standard biomarkers.

## Figures and Tables

**Figure 1:**
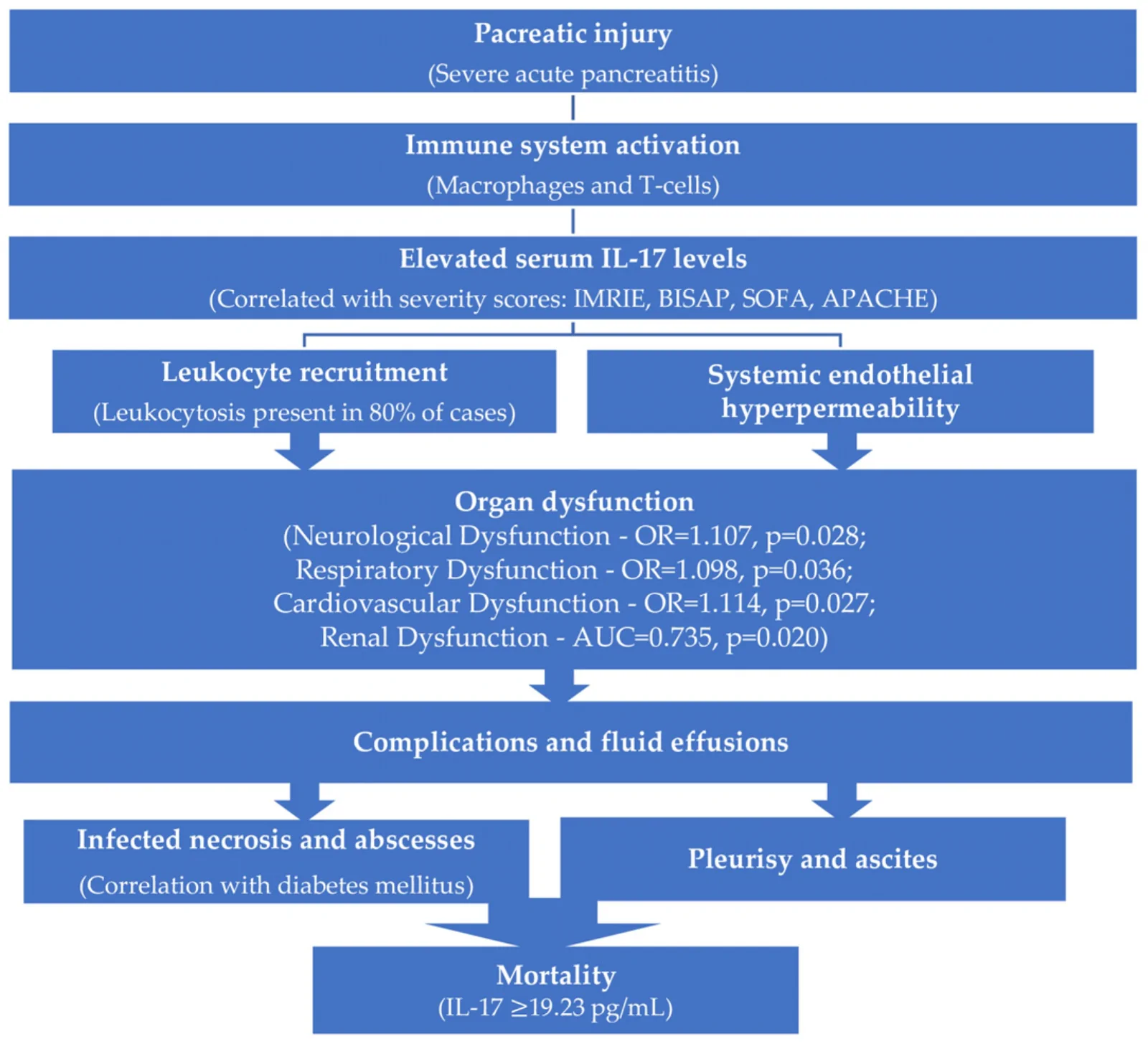
Graphical summary of the role of IL-17 in the inflammatory cascade and clinical outcomes of severe acute pancreatitis.

**Table 1:** Characteristics of the study group.

Characteristics	Severe Acute Pancreatitis (*n* = 35)
Age (median, IQR)	64 (51–76)
Sex female:male	1:2.5
Comorbidities:	
• Neurological	14.29%
• Respiratory	8.57%
• Cardiovascular	42.86%
• Digestive	65.71%
• Renal	14.29%
• Oncological	14.29%
• Diabetes	22.86%
Etiology:	
• Biliary	40%
• Ethanol	45.71%
• Metabolic	20%
• Post-interventional	5.71%
Severity Scores:	
• RANSON (median, IQR)	5 (3–7)
• IMRIE (median, IQR)	4 (2–5)
• BISAP (median, IQR)	3 (2–4)
• SOFA (median, IQR)	4 (2–8)
• APACHE (median, IQR)	11 (5–19)
• SAPS3 (median, IQR)	53 (43–69)

**Table 2:** Complications and dysfunctions of patients with severe acute pancreatitis.

Complications and Dysfunctions	Severe Acute Pancreatitis (*n* = 35)
Complications:	
• Ascites	74.29%
• Pleurisy	60%
• Abscesses	22.86%
• Infected necrosis	22.86%
Dysfunctions:	
• Neurological	31.43%
◯ Seizures	◯ 36.36%
◯ Psychomotor agitation	◯ 36.36%
◯ Behavioral and cognitive alterations	◯ 18.18%
◯ Paresis	◯ 9.2%
• Respiratory	34.29%
◯ Acute respiratory distress syndrome (ARDS)	◯ 75%
◯ Acute pneumonia	◯ 25%
• Cardiovascular	40%
◯ Arrhythmias	◯ 50%
◯ Acute heart failure	◯ 21.42%
◯ Acute myocardial infarction	◯ 14.29%
◯ Pericarditis	◯ 14.29%
• Renal	40%
◯ Acute kidney injury	◯ 85.72%
◯ Acute pyelonephritis	◯ 7.14%
◯ Acute tubular necrosis	◯ 7.14%
• Hematological	45.71%
◯ Thrombocytopenia	◯ 56.25%
◯ Severe anemia	◯ 18.75%
◯ Thrombosis	◯ 12.5%
◯ Disseminated intravascular coagulation	◯ 12.5%
Vasoactive support required	40%

## Data Availability

The original contributions presented in this study are included in the article. Further inquiries can be directed to the corresponding authors.
